# Association between Sleep Quality and Body Composition in Sedentary Middle-Aged Adults

**DOI:** 10.3390/medicina54050091

**Published:** 2018-11-19

**Authors:** Lucas Jurado-Fasoli, Francisco J. Amaro-Gahete, Alejandro De-la-O, Manuel Dote-Montero, Ángel Gutiérrez, Manuel J. Castillo

**Affiliations:** 1 Departament of Medical Physiology, School of Medicine, University of Granada, Av. De la Investigación, 11, 18016 Granada, Spain; amarof@ugr.es (F.J.A.-G.); delao@ugr.es (A.D.-l.-O.); manueldote@correo.ugr.es (M.D.-M.); gutierre@ugr.es (Á.G.); mcgarzon@ugr.es (M.J.C.); 2 PROmoting FITness and Health through physical activity research group (PROFITH), Department of Physical Education and Sports, Faculty of Sport Sciences, University of Granada, Camino de Alfacar, 21, 18071 Granada, Spain

**Keywords:** sleep quality, body composition, body mass index, bone mineral density, lean mass, fat mass

## Abstract

*Background:* Ageing is associated with sleep pattern changes and body composition changes, which are related to several diseases. *Purpose:* This study aimed to analyse the association between sleep quality and an extensive set of body composition parameters (waist-hip ratio, body mass index, bone mineral content, bone mineral density, lean mass, lean mass index, fat mass, fat mass percentage, fat mass index, visceral adipose tissue) and sleep quality in sedentary middle-aged adults. We also aimed to evaluate whether the possible associations accord between subjective and objective measurements of sleep quality. *Methods:* 74 (39 women) middle-aged sedentary adults (40–65 years old) participated in the present study. The sleep quality was assessed using the Pittsburgh sleep quality index (PSQI) scale and accelerometers. A PSQI global score more than 5 indicates poor sleep quality. Weight, height, waist and hip circumferences were measured, and body mass index and waist-hip ratio were also calculated. Body composition was assessed with a dual-energy X-ray absorptiometry scanner. *Results:* The PSQI global score was negatively associated with bone mineral content, bone mineral density, lean mass, lean mass index and positively associated with fat mass percentage. No association was found between accelerometer parameters and body composition variables. *Conclusion:* We showed that a subjective poor sleep quality was negatively associated with bone mineral content (BMC), bone mineral density (BMD), lean mass and lean mass index (LMI) whereas was positively associated with fat mass percentage in middle-aged adults. We also observed that these associations did not accord with objective sleep quality measurements.

## 1. Introduction

Age-related changes in body composition, characterized by a decrease in bone mineral density and muscle mass and an increment of fat mass, is a concern in the aged society [1]. Sarcopenia, obesity and osteopenia/osteoporosis are three frequent chronic diseases in the older population but these conditions are progressive and initiate at a younger age [2,3]. These body composition changes are related to a decrease in the quality of life and an increase of mortality risk [4], producing a significant burden on individual wellness and public health [3].

Another significant concern associated with ageing is sleep pattern changes [5], including a decrease in the quantity and quality of sleep [5]. It has been estimated that approximately 50% of older adults complain about difficulty initiating or maintaining sleep [6]. A poor sleep quality could increase inflammation, decrease melatonin production and disrupt the circadian rhythms [7], which are involved in several diseases related to the ageing process, such as, coronary heart disease [8], type 2 diabetes [9], obesity [10], depression [11], and consequently an increased risk of mortality [6].

Certain studies have proposed a relationship between sleep quality and bone mineral density [12], muscle mass [13] and fat mass [14]. A poor sleep quality deregulates the catabolic/anabolic cycle increasing the risk of osteoporosis [15], induces high insulin resistance increasing the risk of sarcopenia [16], and produces metabolic and endocrine alterations increasing the risk of obesity [17]. However, these studies used subjective questionnaires to assess sleep quality. Although these questionnaires have been previously validated, there are no studies that evaluate the association between sleep quality measured by accelerometry with body composition parameters.

This study aimed to analyse the association between sleep quality (measured using subjective and objective methods) with an extensive set of body composition parameters (waist-hip ratio, body mass index, bone mineral content, bone mineral density, lean mass, lean mass index, fat mass, fat mass percentage, fat mass index, visceral adipose tissue) in sedentary middle-aged adults. We also aimed to evaluate whether these associations were similar considering sleep quality measured by subjective versus objective methods. We hypothesized that participants with poor sleep quality would have low bone mineral density, low lean mass and high fat mass levels. Additionally, we hypothesized that these associations would be similar in subjective and objective methods.

## 2. Materials and Methods

### 2.1. Participants

A total of 74 (39 women) middle-aged sedentary adults (40–65 years old) took part in the present study. The participants were enrolled in the FIT-AGEING study [18], an exercise-based randomized controlled trial (clinicaltrial.gov: ID: NCT03334357). All participants reported to be non-physically active (<20 min of moderate-intensity physical activity on 3 days/week), with stable weight (weight changes <5 kg) over the last 5 months, free of disease, and not taking any medication. The study was approved by the Ethics Committee on Human Research at the University of Granada and Servicio Andaluz de Salud (CEI-Granada) (0838-N-2017) (25 September 2017). The study protocols and experimental design were applied in accordance with the last revised ethical guidelines of the Declaration of Helsinki. All participants signed an informed consent. All variables were assessed at the baseline of the FIT-AGEING study [18].

### 2.2. Sleep Quality Assessment

The sleep quality of the participants was assessed by the Pittsburgh sleep quality index (PSQI) scale, a 19-item scale that provides 7 component scores (ranges 0–3) [19]. It consists of 7 elements: (i) subjective sleep quality (very good to very bad), (ii) sleep latency (≤15 min to >60 min), (iii) sleep duration (≥7 h to <5 h), (iv) sleep efficiency (≥85% to <65% hours sleep/hours in bed), (v) sleep disturbances (not during the past month to ≥ 3 times per week), (vi) use of sleeping medications (none to ≥ 3 times a week), and (vii) daytime dysfunction (not a problem to a very big problem); with a total global score ranging from 0 to 21 [19]. A PSQI global score more than 5 was considered as poor sleep quality [19].

Objective characteristics of sleep-wake patterns were measured with a wrist-worn accelerometer (ActiSleep, ActiGraph, Pensacola, FL, USA) for 7 consecutive days (24 h/day) [18]. The participants received detailed information on how to wear the accelerometer and were asked to remove it only during water activities. They also recorded the times they went to bed each night, woke up each morning and removed the device every day. Data were processed using the ActiLife v. 6.13.3 software (ActiGraph, Pensacola, FL, USA). The accelerometers were initialized to store raw accelerations at a sampling frequency of 100 Hz [20]. The following variables were analyzed: Total sleep time (minutes slept between bedtime and wake time), sleep efficiency (percentage of time asleep while in bed) and wake after sleep onset (minutes awake between sleep onset and wake time). Wake after sleep onset referred to periods of wakefulness occurring after defined sleep onset, and it was a measurement of wakefulness, reflecting sleep fragmentation [21].

### 2.3. Anthropometric and Body Composition Assessment

Body weight and height measurements were performed without shoes and with light clothing, using a pre-validated scale and stadiometer (model 799, Electronic Column Scale, Hamburg, Germany) and the body mass index (BMI) was calculated (weight/height^2^) [22].

We measured (in triplicate) waist circumference (WC) at the midpoint between the lowest rib cage and the top of the iliac crest and hip circumference (HC) around the widest portion of the buttocks with a non-elastic tape (Seca 200, MWS Ltd., Scalesmart, Leicester, UK) to the nearest 0.1 cm. Waist-hip ratio (WHiR) was calculated by dividing waist measurement by hip measurement.

A dual-energy X-ray absorptiometry scanner (Discovery Wi, Hologic, Inc., Bedford, MA, USA) was used to measure body composition following the manufacturer’s recommendations. The whole-body scan was considered to obtain all body composition parameters (bone mineral content, lean mass, fat mass and visceral adipose tissue). We conducted the quality controls, the positioning of the participants, and the analyses of the results following the manufacturer’s recommendations. An automatic delineation of the anatomic regions was performed by the software APEX 4.0.2. We acquired spine phantom quality control scans on each study day. Bone mineral density (BMD) was calculated as bone mineral content (BMC) in g divided by the total bone surface in cm^2^. Lean mass index (LMI) was calculated as lean mass in kg divided by height in m^2^. Similarly, we calculated the fat mass index (FMI) as fat mass in kg divided by height in m^2^. Fat mass was also expressed as weight percentage.

### 2.4. Statistical Analysis

The sample size and power calculations were made based on the data of a pilot study [18]. The Shapiro–Wilk test, visual check of histograms, Q-Q and box plots were used to verify the distribution of all variables. The descriptive parameters were reported as mean and standard deviation.

We conducted simple linear regression models to examine the association of sleep quality (PSQI global score, total sleep time, sleep efficiency and wake after sleep onset) with body composition parameters (BMI, WHiR, BMC, BMD, lean mass, LMI, fat mass, fat mass percentage, FMI, and visceral adipose tissue).

We also conducted multiple linear regression models to test these associations after adjusting by sex, and age.

All analyses were conducted using the Statistical Package for Social Sciences (SPSS, v. 25.0, IBM SPSS Statistics, IBM Corporation) and the level of significance was set at <0.05. Graphical presentations were prepared using GraphPad Prism 5 (GraphPad Software, San Diego, CA, USA).

## 3. Results

The characteristics of the study sample are shown in Table 1.

**Figure 1:**
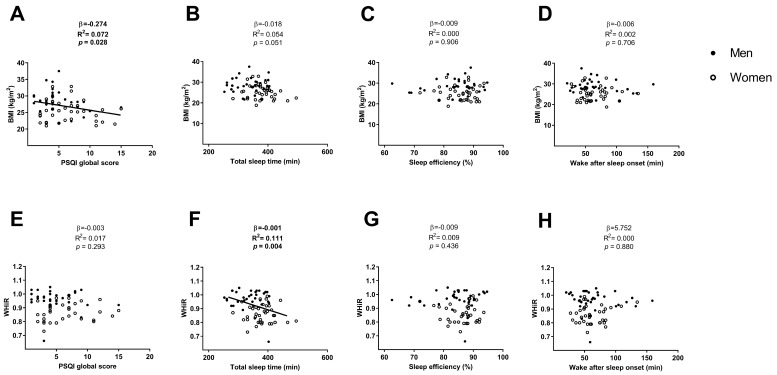
Linear regression graphs between the PSQI global score, total sleep time, sleep efficiency and wake after sleep onset with BMI (Panels **A**, **B**, **C**, and **D**, respectively) and with WHiR (Panels **E**, **F**, **G**, and **H**, respectively). β—non-standardised linear regression coefficient; R^2^—Coefficient of determination; *p* value. Abbreviations: PSQI—Pittsburgh sleep quality index, BMI—body mass index, WHiR—waist-hip ratio.

The PSQI global score was negatively associated with the BMI (β = −0.274, R^2^ = 0.072, *p* = 0.028; Figure 1A), which disappeared after including sex, and both sex and age in the model (all *p* > 0.05; Table 2). The PSQI global score was not associated with the WHiR (β = −0.003, R^2^ = 0.017, *p* = 0.293; Figure 1E) neither after including sex, age, and both sex and age in the model (all *p* > 0.05; Table 2).

No association was found between total sleep time and BMI (β = −0.018, R^2^ = 0.054, *p* = 0.051; Figure 1B), neither including sex or both sex and age in the model (all *p* > 0.05; Table 3) and was negatively associated when we included age (β = −0.019, R^2^ = 0.083, *p* = 0.040; Table 3) in the model. Total sleep time was negatively associated with WHiR (β=−0.001, R^2^ = 0.111, *p* = 0.004; Figure 1F), which disappeared after including sex or both sex and age in the model (all *p* > 0.05; Table 3) and remained after including age in the model (β = −0.001, R^2^ = 0.126, *p* = 0.006; Table 3).

No association was found between sleep efficiency and the wake after sleep onset with BMI and WHiR (all *p* > 0.05; Figure 1C,D, respectively for BMI and Figure 1G,H, respectively for WHiR), neither after including sex age or both sex and age in the model (all *p* > 0.05; Table 4 and Table 5).

**Figure 2:**
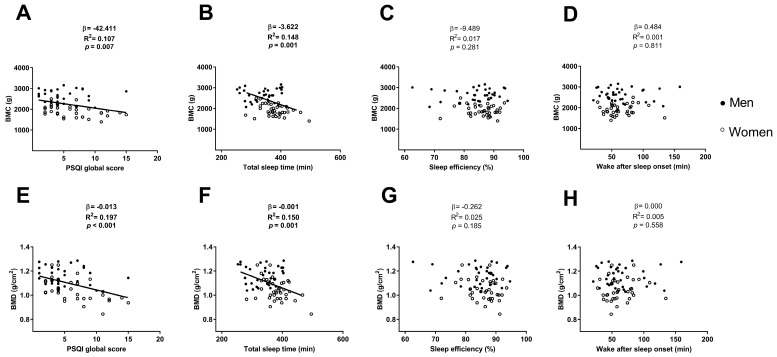
Linear regression graphs between PSQI global score, total sleep time, sleep efficiency and wake after sleep onset with BMC (Panels **A**, **B**, **C**, and **D**, respectively) and with BMD (Panels **E**, **F**, **G**, and **H**, respectively). β—non-standardised linear regression coefficient; R^2^—Coefficient of determination; *p* value. Abbreviations: PSQI—Pittsburgh sleep quality index, BMC—bone mineral content, BMD—bone mineral density.

The PSQI global score was negatively associated with the BMC and the BMD (β = −42.411, R^2^ = 0.107, *p* = 0.007; Figure 2A; β = −0.013, R^2^ = 0.197, *p* < 0.001; Figure 2E respectively), which remained after including sex (β = −20.624, R^2^ = 0.634, *p* = 0.045; β = −0.010, R^2^ = 0.419, *p* = 0.001 respectively; Table 2) and age (β = −42.544, R^2^ = 0.107, *p* = 0.009; β = −0.013, R^2^ = 0.197, *p* < 0.001 respectively; Table 2) in the model. However, when we included both sex and age in the model, the association between the PSQI global score and BMC disappeared (*p* > 0.05; Table 2), and remained with BMD (β = −14.293, R^2^ = 0.230, *p* = 0.003; Table 2).

The total sleep time was negatively associated with the BMC and the BMD (β = −3.622, R^2^ = 0.148, *p* = 0.001; Figure 2B; β = −0.001, R^2^ = 0.150, *p* = 0.001; Figure 2F respectively), which disappeared after including sex in the model or both sex and age in the model (all *p* > 0.05; Table 3) and remained after including age in the model (β = −3.664, R^2^ = 0.152, *p* = 0.001; β = −0.001; R^2^ = 0.159, *p* = 0.001 respectively; Table 3).

No association was found between the sleep efficiency and the wake after sleep onset with the BMC and the BMD (all *p* > 0.05; Figure 2C,D, respectively for BMC and Figure 2G,H, respectively for BMD), neither after including sex, age or both sex and age in the model (all *p* > 0.05; Table 5).

**Figure 3:**
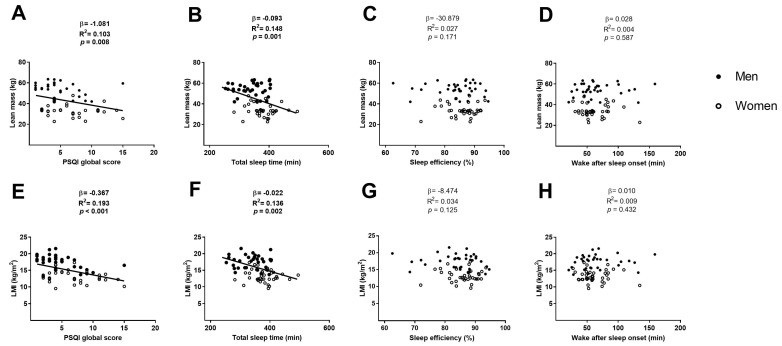
Linear regression graphs between PSQI global score, total sleep time, sleep efficiency and wake after sleep onset with lean mass (Panels **A**, **B**, **C**, and **D**, respectively) and with LMI (Panels **E**, **F**, **G**, and **H**, respectively). β—non-standardised linear regression coefficient; R^2^—Coefficient of determination; *p* value. Abbreviations: PSQI—Pittsburgh sleep quality index, LMI—lean mass index.

The PSQI global score was negatively associated with the lean mass and the LMI (β = −1.081, R^2^ = 0.103, *p* = 0.008; Figure 3A; β = −0.367, R^2^ = 0.193, *p* < 0.001; Figure 3E respectively), which remained after including sex (β = −0.466, R^2^ = 0.725, *p* = 0.044; β = −0.238, R^2^ = 0.640, *p* < 0.001 respectively; Table 2) and age (β = −1.001, R^2^ = 0.115, *p* = 0.016; β = −0.332, R^2^ = 0.228, *p* = 0.001 respectively; Table 2) in the model. However, when we included both sex and age in the model, the association between the PSQI global score and lean mass disappeared (*p* > 0.05; Table 2), and remained with LMI (β = −0.724, R^2^ = 0.219, *p* = 0.004; Table 2).

No association was found between the sleep efficiency and the wake after sleep onset with the lean mass and the LMI (all *p* > 0.05; Figure 3C,D respectively for lean mass Figure 3G,H respectively for LMI), neither when we included sex, age or both sex and age in the model (all *p* > 0.05; Table 4 and Table 5).

**Figure 4:**
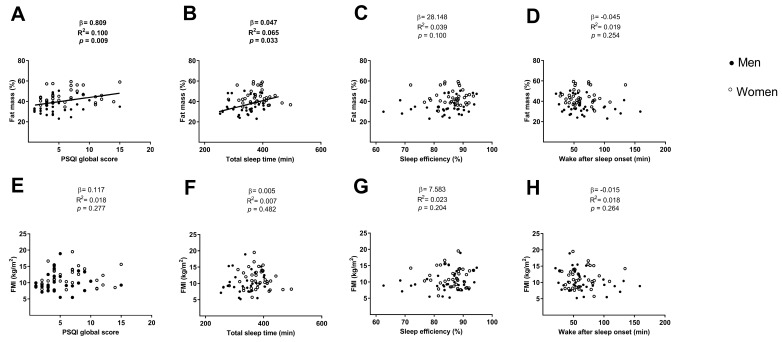
Linear regression graphs between PSQI global score, total sleep time, sleep efficiency and wake after sleep onset with fat mass percentage (Panels **A**, **B**, **C**, and **D**, respectively) and with FMI (Panels **E**, **F**, **G**, and **H**, respectively). β—non-standardised linear regression coefficient; R^2^—Coefficient of determination; *p* value. Abbreviations: PSQI–Pittsburgh sleep quality index, FMI—fat mass index.

The PSQI global score and the total sleep time were positively associated with the fat mass percentage (β = 0.809, R^2^ = 0.100, *p* = 0.009; Figure 4A; β = 0.047, R^2^ = 0.065, *p* = 0.033; Figure 4B, respectively), which disappeared after including sex or both sex and age in the model (all *p* > 0.05; Table 2 and Table 3) and remained after including age in the model (β = 0.765, R^2^ = 0.106, *p* = 0.016; Table 2; β = 0.049, R^2^ = 0.109, *p* = 0.022; Table 3 respectively).

No association was found between the PSQI global score and the total sleep with the FMI (all *p* > 0.05; Figure 4E,F, respectively), neither after including sex, age or both sex and age in the model (all *p* > 0.05; Table 2 and Table 3, respectively).

No association was found between the sleep efficiency and the wake after sleep onset with the fat mass percentage and the FMI (all *p* > 0.05; Figure 4C,D respectively for fat mass percentage and Figure 4G and 4H respectively for FMI), neither when we included sex, age or both sex and age in the model (all *p* > 0.05; Table 4 and Table 5).

## 4. Discussion

The present study showed that a poor subjective sleep quality was negatively associated with BMC, BMD, lean mass and LMI, and positively associated with the fat mass percentage in middle-aged adults. However, no association was found between objective sleep quality and any body composition variable, neither when we accounted for sex and age.

Osteoporosis is a prevalent age-related disease characterized by low bone mass and microarchitectural deterioration of bone tissue [15], and a poor sleep quality could be a risk factor for osteoporosis and osteopenia in middle-aged adults [23]. A poor sleep quality could have different physiological effects that negatively influence the BMD: (i) Alteration of circadian rhythms that disrupt bone microstructure [24]; (ii) an elevation of pro-inflammatory cytokines [25], which are linked to osteoporosis [26]; (iii) an abnormal melatonin secretion [27], since low melatonin levels are related to bone disease and abnormality [28]; (iv) an elevation of cortisol [25], since cortisol hypersecretion decreases bone cell growth and decreases BMD [29]; (v) decreased levels of leptin [25], which adequate levels have been positively correlated with BMD [30]. All aforementioned mechanisms could negatively influence BMD increasing the risk of developing osteoporosis. Our results agree with others studies that demonstrated that poor sleep quality could be associated with low BMD in middle-aged women [12], in young men [31], and older adults [32].

Sarcopenia is the continuous and progressive age-related decline in skeletal muscle mass [16]. A poor quality of sleep has been demonstrated to be a risk factor for age-related sarcopenia [16]. There are several mechanisms that could explain the negative influence of poor sleep quality on lean mass: (i) An increment in the secretion of the catabolic hormone cortisol [33], which is known to stimulate degradation and inhibit synthesis of muscle proteins [34]; (ii) a disruption of the physiological rhythm of the anabolic hormone testosterone; (iii) an alteration of the secretion peak of the anabolic hormone growth hormone [25]; (iv) a decrease in insulin growth factor 1 (IGF-1) concentration [35], which has a key role in the stimulation of muscle protein synthesis [36]; (v) an increment of pro-inflammatory cytokines [25], which are related to muscle atrophy [36]; and (vi) an increase on insulin resistance [37], which may limit the muscle protein synthesis [38]. In relation to lean mass, our results agreed with those obtained by a cross-sectional study that included 1,196 elderly participants [13].

Obesity is characterized by an excessive accumulation of adiposity, which is highly prevalent during the ageing process [39]. A poor sleep quality increases inflammation [25], which is significantly elevated in obesity [40]. Additionally, poor sleep quality alters circadian rhythms increasing fatness through different mechanisms (i.e., dietary habits disruption, hormonal disruption, etc.) [41]. A poor sleep quality can disturb melatonin secretion [27], which has demonstrated to be the key mediator for the optimization of energy balance and body weight regulation [42], with a direct relationship with fatness and obesity [42]. Poor sleep quality is associated with low leptin and high ghrelin, which are likely to increase appetite, and consequently the risk of obesity [25]. It has been reported that low sleep quality decreases adiponectin levels [43], which is inversely correlated with adiposity [44]. Healthy individuals with poor sleep quality are more insulin resistant [37], which is associated with an increment in adiposity [45]. A poor sleep quality through all aforementioned mechanisms could increase adiposity, and consequently, increase the risk of developing obesity. In line with our results, previous studies reported a relationship between poor sleep quality and higher fat mass in middle-aged adults [46], young adolescents [47] and college students [14].

The lack of association between sleep parameters and body composition parameters when sex or age were accounted for, could be explain by: (i) The sex differences in sleep and sleep disorders between women and men [48], which could be based on differences in physiological conditions between sexes like menstrual cycles, male and female hormones [49] and differences in circadian rhythms [50] and (ii) the age-related sleep changes, and the common sleep disturbances that arise with advancing age [6].

Interestingly, we observed different results when the association between sleep quality and body composition was performed considering subjective sleep quality measured by a PSQI questionnaire versus objective sleep quality measured with an accelerometer. A previous study in youth observed that the PSQI and the accelerometer may measure different attributes of sleep, reporting the inadequate capacity of an accelerometry to detect wakefulness, thus lying in bed awake but motionless is likely to be coded as sleep [51]. Due to this limitation, using both complementary assessment methods (objective and subjective) to obtain detailed information beyond the limited data derived from body movements, is recommended [52].

This study has some limitations that have to be considered: Firstly, we cannot clarify by this cross-sectional study whether sleep quality contributes to maintenance adequate body composition variables (i.e., high BMD, low fat mass, and high lean mass), or whether the body composition status has a positive influence on sleep quality. Longitudinal studies are needed to clarify the direction of the association. Secondly, our study only included middle-aged sedentary adults, thus we cannot extrapolate our results to older, younger, and/or physically active individuals. Thirdly, the sample size of this study was relatively small. Finally, the lack of blood parameters mentioned above, does not allow for confirmation that the relationship is due to the proposed physiological mechanisms. Despite the aforementioned limitations, we measured sleep quality subjectively and objectively, which was indeed the strength of this study, since prior similar studies did not objectively measure sleep quality [12,13,14,31,32,46].

## 5. Conclusions

In conclusion, our study showed that a subjective poor sleep quality was negatively associated with BMC, BMD, lean mass and LMI whereas was positively associated with fat mass percentage in middle-aged adults. Interestingly, we observed different results when the association between sleep quality and body composition was performed considering subjective sleep quality measured by a PSQI questionnaire versus objective sleep quality measured with an accelerometer. Nevertheless, further studies are needed to confirm the observed association in individuals with similar and different characteristics since the sample size of this study was relatively small. Longitudinal studies are also required to establish the causal association between body composition variables and sleep quality.

## Figures and Tables

**Table 1:** Descriptive parameters.

	All (*n* = 74)	Men (*n* = 35)	Women (*n* = 39)
Age (years)	53.7 ± 5.1	54.4 ± 5.3	53.0 ± 5.0
**Body composition parameters**			
Body mass index (kg/m^2^)	26.7 ± 3.8	28.3 ± 3.6	25.3 ± 3.3
Waist-Hip ratio	0.91 ± 0.08	0.97 ± 0.07	0.86 ± 0.06
Bone mineral content (g)	2258.1 ± 453.5	2633.6 ± 301.2	1921.1 ± 259.8
Bone mineral density (g/cm^2^)	1.10 ± 0.10	1.16 ± 0.08	1.05 ± 0.09
Lean mass (kg)	43.5 ± 11.7	53.9 ± 6.5	34.1 ± 5.8
Lean mass index (kg/m^2^)	15.2 ± 2.9	17.5 ± 2.0	13.2 ± 1.8
Fat mass (kg)	30.0 ± 8.4	30.9 ± 9.8	29.2 ± 7.1
Fat mass (%)	39.9 ± 9.1	34.7 ± 8.0	44.5 ± 7.4
Fat mass index (kg/m^2^)	10.7 ± 3.1	10.0 ± 3.2	11.4 ± 2.9
**Sleep quality parameters**			
PSQI global score	5.6 ± 3.5	4.8 ± 3.1	6.3 ± 3.6
Total sleep time (min)	359.9 ± 48.8	337.9 ± 46.3	380.1 ± 42.4
Sleep efficiency (%)	85.0 ± 6.3	84.9 ± 7.5	86.1 ± 4.7
Wake after sleep onset (min)	63.9 ± 27.4	65.8 ± 32.4	62.2 ± 22.2

Values are expressed as mean ± standard deviation. Abbreviations: PSQI—Pittsburgh sleep quality index.

**Table 2:** Association of PSQI global score with body composition parameters.

	All (*n* = 74)								
	Model 1			Model 2			Model 3		
Body Composition Parameters	β	R^2^	*p*	β	R^2^	*p*	β	R^2^	*p*
Body mass index (kg/m^2^)	−0.188	0.204	0.112	−0.241	0.094	0.057	−0.144	0.127	0.279
Waist-Hip ratio	−5.498	0.345	0.982	−0.004	0.050	0.178	−1.030	0.111	0.867
Bone mineral content (g)	−20.624	0.634	**0.045**	−42.544	0.107	**0.009**	−0.002	0.144	0.121
Bone mineral density (g/cm^2^)	−0.010	0.419	**0.001**	−0.013	0.197	**<0.001**	−14.293	0.230	**0.003**
Lean mass (kg)	−0.466	0.725	**0.044**	−1.001	0.115	**0.016**	−8.629	0.129	0.259
Lean mass index (kg/m^2^)	−0.238	0.640	**<0.001**	−0.332	0.228	**0.001**	−0.724	0.219	**0.004**
Fat mass (kg)	0.331	0.026	0.255	0.274	0.014	0.346	5.945	0.129	0.256
Fat mass (%)	0.511	0.353	0.058	0.765	0.106	**0.016**	0.083	0.140	0.150
Fat mass index (kg/m^2^)	0.072	0.068	0.506	0.116	0.018	0.297	0.077	0.115	0.588
Visceral adipose tissue (g)	6.407	0.188	0.591	−5.293	0.006	0.687	0.001	0.115	0.583

The analyses were controlled for: Sex (Model 1); age (Model 2); both sex and age (Model 3). β—unstandardized regression coefficient; R^2^, and *p* value were obtained from the multiple linear regression analyses. Abbreviations: PSQI—Pittsburgh sleep quality index; bold values are values that are significant (*p* < 0.05).

**Table 3:** Association of sleep time with body composition parameters.

	All (*n* = 74)								
	Model 1			Model 2			Model 3		
Body Composition Parameters	β	R^2^	*p*	β	R^2^	*p*	β	R^2^	*p*
Body mass index (kg/m^2^)	−0.005	0.182	0.622	−0.019	0.083	**0.040**	−0.876	0.192	0.587
Waist-Hip ratio	0.000	0.391	0.455	−0.001	0.126	**0.006**	−58.250	0.195	0.467
Bone mineral content (g)	−0.441	0.639	0.565	−3.664	0.152	**0.001**	−0.012	0.193	0.532
Bone mineral density (g/cm^2^)	0.000	0.352	0.120	−0.001	0.159	**0.001**	−102.173	0.219	0.110
Lean mass (kg)	−0.004	0.734	0.825	−0.096	0.186	**0.001**	0.000	0.190	0.718
Lean mass index (kg/m^2^)	−0.003	0.563	0.544	−0.023	0.237	**<0.001**	−2.916	0.198	0.393
Fat mass (kg)	0.007	0.019	0.775	−0.003	0.010	0.870	0.000	0.190	0.765
Fat mass (%)	0.006	0.275	0.774	0.049	0.109	**0.022**	0.261	0.190	0.724
Fat mass index (kg/m^2^)	−7.391	0.039	0.993	0.006	0.016	0.451	0.020	0.189	0.991
Visceral adipose tissue (g)	−0.576	0.221	0.548	−2.049	0.073	**0.033**	−0.009	0.193	0.555

The analyses were controlled for: Sex (Model 1); age (Model 2); both sex and age (Model 3). β—unstandardized regression coefficient; R^2^, and *p* value were obtained from the multiple linear regression analyses. Bold values are values that are significant (*p* < 0.05).

**Table 4:** Association of sleep efficiency with body composition parameters.

	All (*n* = 74)								
	Model 1			Model 2			Model 3		
Body Composition Parameters	β	R^2^	*p*	β	R^2^	*p*	β	R^2^	*p*
Body mass index (kg/m^2^)	3.686	0.183	0.584	−1.190	0.024	0.869	0.120	0.035	0.598
Waist-Hip ratio	0.020	0.386	0.879	−0.122	0.028	0.460	1.861	0.031	0.869
Bone mineral content (g)	71.598	0.637	0.896	−960.826	0.019	0.279	0.000	0.031	0.918
Bone mineral density (g/cm^2^)	−0.101	0.332	0.545	−0.266	0.031	0.180	−5.737	0.036	0.527
Lean mass (kg)	−2.926	0.734	0.807	−32.049	0.057	0.152	−0.053	0.033	0.716
Lean mass index (kg/m^2^)	−2.532	0.564	0.502	−8.966	0.123	0.092	−0.435	0.043	0.365
Fat mass (kg)	23.375	0.047	0.150	20.077	0.032	0.212	0.132	0.061	0.148
Fat mass (%)	15.583	0.286	0.298	29.174	0.079	0.044	0.116	0.049	0.261
Fat mass index (kg/m^2^)	6.054	0.053	0.313	7.752	0.032	0.196	0.254	0.046	0.306
Visceral adipose tissue (g)	148.816	0.217	0.827	−342.253	0.011	0.651	0.000	0.031	0.824

The analyses were controlled for: Sex (Model 1); age (Model 2); both sex and age (Model 3). β—unstandardized regression coefficient; R^2^, and *p* value were obtained from the multiple linear regression analyses. Bold values are values that are significant (*p* < 0.05).

**Table 5:** Association of wake after sleep onset with body composition parameters.

	All (*n* = 74)								
	Model 1			Model 2			Model 3		
Body Composition Parameters	β	R^2^	*p*	β	R^2^	*p*	β	R^2^	*p*
Body mass index (kg/m^2^)	−0.010	0.184	0.502	−0.006	0.025	0.739	−0.634	0.011	0.528
Waist-Hip ratio	−7.338	0.386	0.807	4.170	0.020	0.912	−13.219	0.006	0.791
Bone mineral content (g)	−0.414	0.637	0.737	0.509	0.003	0.803	−0.004	0.007	0.771
Bone mineral density (g/cm^2^)	0.000	0.329	0.745	0.000	0.010	0.546	14.698	0.007	0.714
Lean mass (kg)	0.004	0.734	0.897	0.031	0.034	0.551	0.186	0.007	0.772
Lean mass index (kg/m^2^)	0.005	0.563	0.580	0.011	0.096	0.365	1.764	0.016	0.406
Fat mass (kg)	−0.055	0.049	0.136	−0.053	0.040	0.151	−0.604	0.039	0.132
Fat mass (%)	−0.034	0.285	0.321	−0.047	0.023	0.224	−0.506	0.023	0.269
Fat mass index (kg/m^2^)	−0.014	0.054	0.307	−0.016	0.027	0.253	−1.152	0.022	0.294
Visceral adipose tissue (g)	−0.510	0.218	0.741	−0.107	0.008	0.951	−0.003	0.007	0.735

The analyses were controlled for: Sex (Model 1); age (Model 2); both sex and age (Model 3). β—unstandardized regression coefficient; R2, and *p* value were obtained from the multiple linear regression analyses.
